# Design and statistical analysis reporting among interrupted time series studies in drug utilization research: a cross-sectional survey

**DOI:** 10.1186/s12874-024-02184-8

**Published:** 2024-03-09

**Authors:** Yuanjin Zhang, Yan Ren, Yunxiang Huang, Minghong Yao, Yulong Jia, Yuning Wang, Fan Mei, Kang Zou, Jing Tan, Xin Sun

**Affiliations:** 1 Chinese Evidence-Based Medicine Center, West China Hospital, Sichuan University, 37 Guo Xue Xiang, Chengdu, 610041 Sichuan China; 2 NMPA Key Laboratory for Real World Data Research and Evaluation in Hainan, Chengdu, China; 3 Sichuan Center of Technology Innovation for Real World Data, Chengdu, China; 4 Hainan Healthcare Security Administration Key Laboratory for Real World Data Research, Chengdu, China

**Keywords:** Drug utilization, Interrupted time series, Pharmacoepidemiology, Quasi-experimental design, Segmented regression

## Abstract

**Introduction:**

Interrupted time series (ITS) design is a commonly used method for evaluating large-scale interventions in clinical practice or public health. However, improperly using this method can lead to biased results.

**Objective:**

To investigate design and statistical analysis characteristics of drug utilization studies using ITS design, and give recommendations for improvements.

**Methods:**

A literature search was conducted based on PubMed from January 2021 to December 2021. We included original articles that used ITS design to investigate drug utilization without restriction on study population or outcome types. A structured, pilot-tested questionnaire was developed to extract information regarding study characteristics and details about design and statistical analysis.

**Results:**

We included 153 eligible studies. Among those, 28.1% (43/153) clearly explained the rationale for using the ITS design and 13.7% (21/153) clarified the rationale of using the specified ITS model structure. One hundred and forty-nine studies used aggregated data to do ITS analysis, and 20.8% (31/149) clarified the rationale for the number of time points. The consideration of autocorrelation, non-stationary and seasonality was often lacking among those studies, and only 14 studies mentioned all of three methodological issues. Missing data was mentioned in 31 studies. Only 39.22% (60/153) reported the regression models, while 15 studies gave the incorrect interpretation of level change due to time parameterization. Time-varying participant characteristics were considered in 24 studies. In 97 studies containing hierarchical data, 23 studies clarified the heterogeneity among clusters and used statistical methods to address this issue.

**Conclusion:**

The quality of design and statistical analyses in ITS studies for drug utilization remains unsatisfactory. Three emerging methodological issues warranted particular attention, including incorrect interpretation of level change due to time parameterization, time-varying participant characteristics and hierarchical data analysis. We offered specific recommendations about the design, analysis and reporting of the ITS study.

**Supplementary Information:**

The online version contains supplementary material available at 10.1186/s12874-024-02184-8.

## Introduction

Drug utilization research has received substantial attention from health researchers and policymakers in recent years. Interventions in drug utilization research may range from clinical guideline publications to drug programmes or policies. The randomized controlled trial is considered as the gold standard design for evaluating the causal effect of an intervention. Nevertheless, it is not always feasible or ethical in the field, as these interventions are often targeted at population level [1–4]. As a strong quasi-experimental design, interrupted time series (ITS) design has increasingly been used for the evaluation of drug utilization interventions by comparing the level and trend of outcomes after intervention with the pre-intervention underlying level and trend [5–10].

Several important methodological issues need to be considered when conducting ITS studies, such as time period selection, sample size, missing data, autocorrelation, and non-stationary and seasonality, which have been described in previous tutorials [5, 11–15]. Three issues newly emerging in recent years also require additional methodological considerations. First, the correct setting and interpretation of the ITS regression model should be underlined. In a particular ITS model setting including parameters of level change and slope change, β2 represents the immediate level (or intercept) change immediately following the intervention [16]. However, some current peer-reviewed studies conducted the wrong ITS model but still described β2 as the level change at the time of interruption, which will lead to an erroneous and biased result for the main effect of the immediate level change of the time-series (see details in Appendix 1) [17]. Second, it is possible that the participants’ characteristics are not constant at different time points. The ITS method might be affected by time-varying confounding, which may result in a misleading finding [5, 6]. Third, heterogeneity in clusters should be appropriately addressed in ITS studies if the dataset contains a hierarchical structure and has within and/or between cluster heterogeneity [18–20]. A study pointed out that authors need to consider this issue and use appropriate analysis methods such as mixed-effect model [19]. However, since not all articles contain multiple-level data, the proportion of studies that have not yet addressed this issue remains unclear.

Previous studies might not have comprehensively addressed these methodological issues [11, 21–25]. The last survey on the ITS studies in drug utilization research was published in 2015 and did not cover the new methodological issues mentioned above [21]. Additionally, despite increasing tutorials for conducting ITS have been published in recent years, it is still unclear whether the quality of current ITS studies in drug utilization has improved. Thus, we conducted a cross-sectional survey among the published ITS studies in drug utilization, aiming to identify the potential methodological gaps and give suggestions for improvement.

## Methods

### Eligibility criteria

We included empirical studies that used ITS design and focused on intervention related to drug utilization, with no limitation to study population or types of outcomes. The definition of ITS study was followed as the previous methodological studies, as “a time series of a particular outcome of interest is used to establish an underlying trend, which is ‘interrupted’ by an intervention at a known point in time” [4, 5]. We focused on the ITS study about drug utilization, whose intervention was about various medical, social and economic aspects of drug use [26].

Studies meeting any of the following criteria were excluded: (1) letters, commentaries, study protocols, conference abstracts, systematic reviews, meta-analyses, randomized controlled studies; (2) not written in English; (3) a methodological paper with an ITS example; (4) ITS analysis was not the main result.

### Search strategy

We searched PubMed in January 2022 for ITS studies published in 2021. We used Mesh terms and text words correlated to interrupted time series to develop the search strategy, including “interrupted time series”, “change point”, “segmented regression”, “repeated measures study” and so on. The details of the search strategy are presented in Appendix 2.

### Study process

A structured, pilot-tested checklist was developed to screen titles, abstracts, and full texts for potentially eligible studies, using prespecified eligibility criteria. Two researchers (YZ and YH), who were trained in epidemiology and biostatistics with sufficient experiences in ITS analysis, conducted the records screening independently. Any disagreements were resolved by the discussion and adjudication by a third reviewer (YR).

Before informally extracting the data, two researchers (YZ and YH) randomly selected 15 (10%) eligible studies and extracted the data independently. They checked for consistency, and any disagreements were adjudicated by a third reviewer (YR). In total, the agreement between the two researchers (YZ and YH) was above 95%. Then a single researcher (YZ) extracted the further 138 studies.

### Development of the data extraction form

 A structured questionnaire was developed to investigate the design and analysis characteristics of ITS studies on drug utilization research. Initially, we reviewed the published methodological literature and statements to design the initial data extraction form [5, 21–25]. Then, we invited four experts (XS, RY, JT and MY) in clinical epidemiology and biostatistics to review and discuss the data extraction form, assessing the relevance and applicability of candidate items. We randomly selected 30 studies as pilot extraction to check if there were any items inappropriate.

Finally, we identified three parts of the design and analysis characteristics of ITS studies, including (1) general characteristics, (2) design, and (3) statistical analysis. The detailed items of the data extraction form are shown in Appendix 3.

### Data analysis

All items in the data extraction form were summarized using descriptive statistics. For categorical variables, we presented frequencies and percentages; for continuous variables, we presented mean with standard deviation (SD) or median with interquartile range (IQR). All statistical analyses were conducted using Stata.15.1.

## Results

**Fig. 1:**
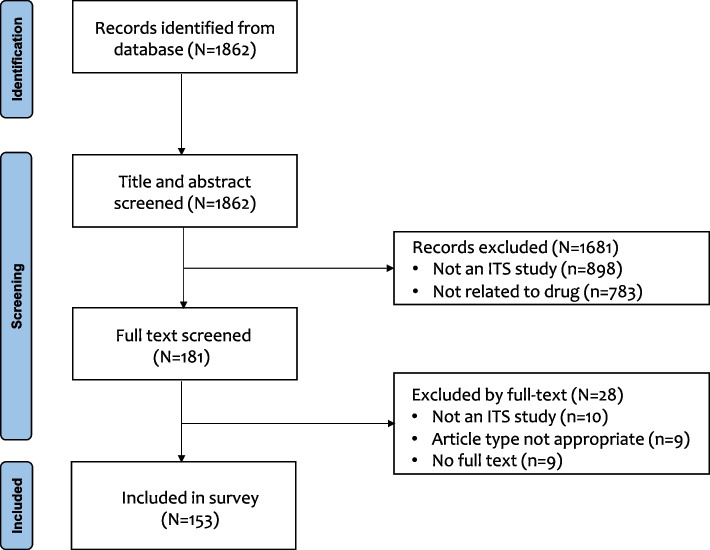
Flow diagram of the selection results

Through the search from PubMed, we identified 1862 records. After reviewing titles, abstracts and full texts, 153 studies were finally included in our analysis (Fig. 1). Appendix 4 shows the details of all included studies.

### General characteristics

Of the 153 included studies, 70.6% (108/153) were multi-site studies (Table 1). Hospital data (39.2%, 60/153), insurance databases (17.6%, 27/153) and other administrative databases (35.3%, 54/153) were the three most common data sources of the included ITS studies.

**Table 1:** General characteristics of included ITS studies (N=153)

Characteristics	*n*	%
**Study sites**		
Multi-sites	108	70.6
Single-site	45	29.4
**Data source**^**a**^		
Hospital data (from electronic medical record)	60	39.2
Other administrative databases	54	35.3
Insurance (claims) database	27	17.6
Others^b^	12	7.8
**Intervention stages**		
Single	126	82.4
Multiple	27	17.6
**Type of intervention**		
Prescription restriction	45	29.4
Drug price change (price, purchase or reimbursement policy)	27	17.6
Clinical guideline change (guidelines, statements or publications)	23	15.0
Education or training for physicians	22	14.4
Digital technology	13	8.5
Drug safety advisory	13	8.5
Drug approval or withdrawal	8	5.2
Others^c^	2	1.3
**Level of intervention**		
National	90	58.8
Hospital	30	19.6
State or province	21	13.7
Region	6	3.9
Others^d^	6	3.9
**Measure type of outcome**		
Drug utilization	125	81.7
Health outcomes	17	11.1
Expenditures	10	6.5
Others^e^	1	0.7
**Data type of outcome**		
Rate	98	64.1
Continuous	38	24.8
Count	15	9.8
Binary	2	1.3

^a^Administrative medical databases are massive repositories of data collected in healthcare for various purposes. Such databases are maintained in hospitals, health maintenance organizations and health insurance organizations. In this article, other administrative databases in this article included the administrative database which was not from hospital or health insurance organizations

^b^Others included registry (6 studies), survey (3 studies), statistical yearbook (2 studies) and cohort study (1 study)

^c^Others included drug shortage (1 study) and change drug packaging (1 study)

^d^Others included Europe (4 studies), city (1 study) and community (1 study)

^e^Others included cannabis-related criminal offences (1 study)

For the intervention characteristics, 82.4% (126/153) analyzed the single-stage intervention. The prescription restriction was the most common intervention in the included studies (29.4%, 45/153). Drug price change and clinical guideline change were the next two most common interventions (17.6%, 27/153 and 15.0%, 23/153). And the interventions were mainly implemented at the national level (58.8%, 90/153).

For the outcome characteristics, the most common outcomes were drug utilization (81.7%,125/153), and some studies focused on health outcomes (11.1%, 17/153) and expenditures (6.5%, 10/153). Rate (64.1%, 98/153), continuous (24.8%, 38/153), and count (9.8%, 15/153) were the three most common data types of outcomes.

### Study design

#### Rationale for ITS design

Among the included studies, only 28.1% (43/153) reported the rationale for using ITS design (Table 2). All studies gave a clear segment time of the intervention. There were 12.4% (19/153) studies that used ITS design with the control group to strengthen the validity of the study design.

**Table 2:** Design characteristics (rationale for ITS, data handling and model structure) of included studies

Characteristics	*n* or median	% or IQR
**Rationale for ITS**		
**Did the author give the reason/rationale for using ITS study design? (*N* = 153)**		
Yes	43	28.1
**Clearly segment time? (*N* = 153)**		
Yes	153	100.0
**Use of control group (*N* = 153)**		
Yes	19	12.4
**Type of control (*N* = 19)**^**a**^		
Characteristic	11	57.9
Location	6	31.6
Historical	1	5.3
Outcome	1	5.3
**Data collection and aggregation**		
**Data collection (*N* = 153)**		
Retrospective	141	92.2
Prospective	12	7.8
**For studies used prospective data, if they have pre-specified protocol? (*N* = 12)**		
Yes	2	16.7
**Raw data (*N* = 153)**		
Contained individual-level data	127	83.0
Only contained aggregated-level data	26	17.0
**Analysis unit **^**b**^** (*N* = 153)**		
Aggregated unit	149	97.4
Individual unit	4	2.6
**Time intervals used for ITS with aggregated unit (*N* = 149)**		
Month	110	73.8
Quarter	17	11.4
Week	7	4.7
Year	6	4.0
Day	4	2.7
Six-month	2	1.3
Two-week	2	1.3
Two-month	1	0.7
**Number of time points (*N* = 149)**^**c**^	48	30, 72
**Did the author give the rationale for the number of time points? (*N* = 149)**		
Yes	31	20.8
**The ITS model structure**		
**Type of ITS impact model (*N* = 153)**		
Level and slope change	139	90.8
Level change only	6	3.9
Slope change only	6	3.9
Unclear	2	1.3
**Did the author give the rationale for using this model? (*N* = 153)**		
Yes	21	13.7
**Did the author allow for a delay impact? (*N* = 153)**		
Yes	42	27.5
**Methods of dealing with delay (*N* = 42)**^**d, e**^		
Sensitivity	19	45.2
Segment	11	26.2
Excluded	10	23.8
Delay	6	14.3
**Did the author give the rationale for setting the transition period? (*N* = 42)**		
Yes	23	54.8

^a^Location: use a different area as control; Outcome: use an outcome not affected by the intervention as control; Characteristic: use a group not targeted by an intervention as control; Historical: compare a previous group to a current group

^b^Aggregated data refer to summary statistics (e.g., mean, percentage, median) calculated across individual data

^c^We reported the median and interquartile range (25% and 75%)

^d^Delay: where the delay was acknowledged and included in pre- or post-interruption segment; Excluded: where a separate segment was used for the delay time period, but this was excluded from the analysis; Segment: where a separate segment was used for the delay time period, and this was included in analysis; Sensitivity: where the delay was modelled as part of a sensitivity analysis, but ignored in main analysis)

^e^Some studies used more than one method to deal with delay

#### Data collection and aggregation

Of the 153 studies, most of the included studies (92.2%, 141/153) collected data retrospectively. In 12 studies used prospective data, and only 16.7% (2/12) had pre-specified study protocol. In total, 83.0% (127/153) contained individual-level data in the raw dataset, and only 17.0% (26/153) collected aggregated-level data. Most studies (97.4%, 149/153) used the aggregate unit as the ITS analysis unit, and only 2.6% (4/153) used the individual unit.

For the 149 studies with the aggregated unit, the most common time interval was monthly (73.8%, 110/149). The median (IQR) of total time points was 48 (30, 72). Only 20.8% (31/149) clarified the rationale for the number of time points (sample size calculation).

#### The ITS model structure

Of 153 included studies, 90.8% (139/153) set the ITS model structure including both level change and slope change, 3.9% (6/153) included level change only and 3.9% (6/153) included slope change only. However, only 13.7% (21/153) gave the rationale for using this model structure. 27.5% (42/153) studies considered the potential delay effects, and 54.8% (23/153) of them reported the rationale for setting the transition period.

### Statistical analysis

#### Basic statistical analysis characteristics

Various statistical methods were used to analyze the ITS studies (Table 3). Among the total 153 studies, OLS (30.7%, 47/153) and ARIMA (15.7%, 24/153) were the two most popular methods of the ITS regression model. However, 15.0% (23/153) did not report the regression model.

**Table 3:** Basic statistical characteristics of included ITS studies

Characteristics	*n*	%
**Regression model **^**a**^** (*N* = 153)**		
OLS	47	30.7
ARIMA	24	15.7
GLS	13	8.5
OLS with Newey-West standard errors	12	7.8
Poisson	12	7.8
Mixed effect model	8	5.2
Logistic	4	2.6
Generalized estimating equation	4	2.6
Weighted least square regression	2	1.3
Others ^b^	4	2.6
Unclear	23	15.0
**Other statistical analysis characteristics**		
**Did the author consider missing data? (*N* = 153)**		
Yes	31	20.3
**Sensitivity analysis (*N* = 153)**		
Yes	49	32.0
**Methods for sensitivity analysis (*N* = 49)** ^c, d^		
Transition period	19	38.8
Change measurement of outcomes	10	20.4
Change measurement of study population	11	22.5
Change ITS model setting	9	18.4
Add covariates	2	4.1
Other	11	22.4
**Statistical software (*N* = 153)**		
SAS	54	35.3
Stata	48	31.4
R	25	16.3
SPSS	5	3.3
Not report	21	13.7
**Data availability (*N* = 153)**		
Yes	3	2.0
**Code availability (*N* = 153)**		
Yes	5	3.3

^a^This item refers to the statistical method for the main results in a study

^b^Others included fixed effect model (1 study), negative binomial model (1 study), quasi-poisson model (1 study) and linear probability model (1 study)

^c^Transition period: change the interrupted time or time period in the regression model; Change ITS model setting: change the ITS impact model (e.g., from both level and slope change to only level change)

Some studies used more than one method for sensitivity analysis

Of these 153 studies, 31 studies addressed missing data and 49 studies did the sensitivity analysis. Most studies (86.3%, 132/153) reported the software for statistical analysis, in which SAS (35.3%, 54/153) and Stata (31.4%, 48/153) were the two most popular software for ITS analysis. Only 3.3% (5/153) studies uploaded the full code and 2.0% (3/153) shared the datasets.

#### Basic methodological considerations (Autocorrelation, non-stationary and seasonality)

Among the 149 studies with aggregated-level outcome and time series data, 14 studies considered all of the three methodological issues of time series data (Table 4). 117 studies considered at least one of three methodological issues. Specifically, autocorrelation was acknowledged in 108 studies, non-stationarity was acknowledged in 20 studies, and seasonality was acknowledged in 60 studies. Among the studies adjusted for autocorrelation, non-stationary and seasonality, 25.0% (27/108), 5% (1/20) and 16.7% (10/60) respectively failed to specify the methods they used.

**Table 4:** Characteristics of the basic methodological considerations (autocorrelation, non-stationarity, seasonality) (Only for ITS with aggregated unit)

Characteristics	*n*	%
**Considered all of three methodological issues (autocorrelation, non-stationarity and seasonality) (*N* = 149)**		
Yes	14	9.4
**Considered at least one of three methodological issues (*N* = 149)**		
Yes	117	78.5
**Autocorrelation**		
**Autocorrelation acknowledged (*N* = 149)**		
Yes	108	72.5
**Autocorrelation acknowledged (ITS study used ARIMA model) (*N* = 24)**		
Yes	20	83.3
**Autocorrelation acknowledged (ITS study used Non-ARIMA model) (*N* = 125)**		
Yes	88	70.4
**Autocorrelation identified methods (*N* = 108)**		
Durbin Watson test	40	37.0
ACF	9	8.3
Cumby-Huizinga test	5	4.6
Ljung-Box2 test	3	2.8
Others ^a^	4	3.7
Not reported	47	43.5
**Adjusted for autocorrelation (*N* = 108)**		
Yes	65	60.2
No adjustment for autocorrelation (after statistical test)	16	14.8
Unclear	27	25.0
**If yes, which method was used? (*N* = 65)**		
ARIMA	31	47.7
GLS	16	24.6
OLS with Newey-West standard errors	12	18.5
Add lag terms	5	7.7
Generalized Estimating Equation	1	1.5
**Non-stationarity**		
**Non-stationarity acknowledged (*N* = 149)**		
Yes	20	13.4
**Non-stationarity acknowledged (ITS study used ARIMA model) (*N* = 24)**		
Yes	11	45.9
**Non-stationarity acknowledged (ITS study used Non-ARIMA model) (*N* = 125)**		
Yes	9	7.2
**Non-stationarity identified methods (*N* = 20)**		
Augmented Dickey-Fuller test	8	40.0
Plot the raw data	1	5.0
Not reported	11	55.0
**Adjusted for non-stationarity (*N* = 20)**		
Yes	15	75.0
No adjustment for non-stationarity (after statistical test)	4	20.0
Unclear	1	5.0
**If yes, which method was used? (*N* = 15)**		
ARIMA	11	73.3
Others ^b^	4	26.7
**Seasonality**		
**Seasonality acknowledged (*N* = 149)**		
Yes	60	40.3
**Seasonality acknowledged (ITS study used ARIMA model) (*N* = 24)**		
Yes	15	62.5
**Seasonality acknowledged (ITS study used Non-ARIMA model) (*N* = 125)**		
Yes	45	36.0
**Seasonality identified methods (*N* = 60)**		
Augmented Dickey-Fuller test	4	6.7
Plot the raw data	3	5.0
Others ^c^	6	10.0
Not reported	47	78.3
**Adjusted for seasonality (*N* = 60)**		
Yes	41	68.3
No adjustment for seasonality (after statistical test)	9	15.0
Unclear	10	16.7
**If yes, which method was used? (*N* = 41)**		
Add seasonality terms	18	43.9
ARIMA	13	31.7
Fourier function	8	19.5
Others^d^	2	4.9

^a^Others included residual plots (2 studies), Bartlett formula (1 study), Breusch-Godfrey test (1 study)

^b^Others included add dummy variable (3 studies) and first difference (1 study)

^c^Others included Cumby-Huizinga test (1 study), Kruskal–Wallis test (1 study), Webel-Ollech overall seasonality (1 study), Summary statistics (1 study), add seasonality terms (1 study), test lagged correlation (1 study)

^d^Others included Holt-Winters seasonal smoothing approach (1 study) and Lag period (1 study)

We also compared the differences in studies that used the ARIMA model and the non-ARIMA model. The results showed that the studies that used the ARIMA model were more likely to consider the potential autocorrelation (83.3%, 20/24 vs 70.4%, 88/125), non-stationary (45.9%, 11/24 vs 7.2%, 9/125) and seasonality (62.5%, 15/24 vs 36.0%, 45/125).

### Additional methodological considerations

#### Incorrect interpretation of level change due to time parameterization

Of the 153 studies, only 39.2% (60/153) reported the specific regression model and interpreted the coefficients in the article or supplementary files (Table 5). Moreover, we found that 15 studies gave incorrect interpretations of level change due to time parameterisation. To be more specified, these studies reported the model as “${Y}_{t}={\beta }_{0}+{\beta }_{1}{T}_{t}+{\beta }_{2}{X}_{t}+{\beta }_{3}{T}_{t}\bullet {X}_{t}$”, which included parameters for level change and slope change. But they described ${\beta }_{2}$ as “level change at the time of interruption. As we discussed in Appendix 1, this will lead to an incorrect result for the effect of the immediate level change if the study used this model for statistical analysis.

**Table 5:** Additional methodological considerations (parameters setting, individual-level covariates and hierarchical data structure) for ITS studies

Characteristics	*n*	%
**Incorrect interpretation of level change due to time parameterization **^**a**^		
**Reported the regression model and interpreted the coefficients (*N* = 153)**		
Yes	60	39.2
**Where did the author report the regression model and the interpretation of coefficients? (*N* = 60)**		
In article	47	78.3
In supplementary material	13	21.7
**The interpretation of level change due to time parameterization was incorrect **^**b**^		
Yes	15	-
**Individual-level characteristics**		
**Has individual-level data (*N* = 153)**		
Yes	127	83.0
**Consider individual-level characteristics (*N* = 127)**		
Yes	24	18.9
**How to control individual-level characteristics (*N* = 24) **^**c**^		
Add covariates	21	87.5
Stratified Analysis	7	29.2
Other	4	16.7
**Hierarchical data structure**		
**Data structure for ITS analysis (*N* = 149)**^**d**^		
Hierarchical data (more than one level) ^e^	97	65.1
**Whether the author handled hierarchical data (*N* = 97)**		
Yes	23	23.7
**Methods for handling hierarchical data (*N* = 23)**		
Stratified by sites	13	56.5
Mixed effect model	6	26.1
Generalized estimating equation	2	8.7
Fixed-effect model	1	4.3
Two-stage analysis	1	4.3
**Considered cluster effects in which level? (*N* = 23)**		
Hospital/clinic/other healthcare provider	15	65.2
Province/State/Region	4	17.4
Nation	2	8.7
Unclear	2	8.7
**Whether the author reported the differences across sites (*N* = 23)**		
Yes	16	69.6
**If yes, how to present the differences across site? (*N* = 16)**		
Figure	7	43.8
Both table and figure	6	37.5
Table	3	18.8

^a^If the researchers set an ITS model with both level change and slope change, and used the product between their calendar time variable and the indicator variable indicating pre- versus post-intervention time periods to represent the post-intervention linear segment, then the interpretation was wrong (More details in Appendix 1)

^b^For this item, we did not calculate the proportion as the denominator is difficult to define. We believe that using either 60 (the number of studies reporting regression models) or 139 (the number of models including level change and slope change) as the denominator would be inappropriate

^c^Some studies used more than one method to control individual-level characteristics

^d^This part only included ITS studies with aggregated analysis units (*n* = 149) because the mishandling of data hierarchy only takes place in the ITS study with aggregated analysis unit

^e^For the studies that contained individual-level data, we calculated how many levels are there in the dataset excluded individual data (which cannot be repeated measured). For example, the raw data was a three-level hierarchy of patient, hospital and region and the repeated measured level were hospital and region. We defined this dataset as a two-level hierarchical data for ITS analysis. For the studies that only contained aggregated data, we calculated how many levels are there in the dataset directly

#### Individual-level characteristics

Among 153 included studies, 83.0% (127/153) contained individual-level data in the raw dataset. Of these 127 ITS studies, 18.9% (24/127) considered individual-level characteristics.

#### Handling hierarchical data

Of the 149 studies that conducted aggregated unit ITS analysis, 65.1% (97/149) studies included hierarchical data. However, only 23.7% (23/97) of them considered this hierarchical structure. In further analyzing these 23 studies, stratified analysis (56.5%, 13/23) was the most common method to address the hierarchical structure of data, mixed-effect model (26.1%, 6/23) and generalized estimating equation (8.7%, 2/23) were the next two common methods. Authors usually considered the hospital-level cluster effect (65.2%, 15/23), and 17.4% (4/23) studies considered the cluster effects in provinces. Additionally, 69.6% (16/23) estimated and reported the differences across sites.

## Discussions

### Findings and interpretations

This study provides updated evidence on the quality of ITS studies and found that most ITS studies in drug utilization fail to consider the methodological issues of design and statistical analysis comprehensively.

Three main issues of ITS study design need to be considered. First, most studies did not give the rationale for using ITS design. Although it is an appropriate method when randomization is not feasible, the basic ITS design may be affected by confounding due to co-interventions or other events occurring around the study period [27, 28]. Thus, we recommended that the author should give the rationale for using ITS design, such as for ethical consideration or no adequate control group. Second, most of the studies did not report the consideration of the study period, time interval and sample size. The selection of time period should be a balance between statistical requirements and research problem-driven decisions [29, 30]. A simulation study found that sample size per time point had a large impact on power in ITS study. Even though the studies meet the requirement of minimum time points, most analyses were underpowered if the sample size per time point was low [30]. Therefore, the author should balance the number of time points and the sample size per time point. Meanwhile, if the period is too short, there may be too little data to model the trend. However, if the period is too long, it may be affected by historical bias. Third, most of the studies used the ITS model structure with both level change and slope change. However, only a few studies analyzed the intervention (whether it will lead to immediate change or sustained change) and chose the ITS model structure to fit it well [5, 29]. When the model was misspecified, the results of ITS were not robust anymore [31].

Meanwhile, we found five issues that may affect the quality of statistical analysis. First, most of the studies did not mention the missing data. A study mentioned that most of the study used data aggregated at the population level, but it will lead to bias when data are missing at random at the individual level [11]. In a simulated scenario in this study, if the outcome is missing at random for male but is fully observed for female, the aggregated data will show a wrong seasonal pattern. Second, the considerations of autocorrelation, non-stationary and seasonality are still poor among current ITS studies. Ignoring the characteristics of time series data may not provide robust results [5]. Third, more than half studies did not report the regression model, which might lead to an unclear understanding of statistical methods for readers. Moreover, for the studies that reported the regression model, 15 studies used the setting “${TX}_{t}$” instead of “${({T-T}_{0})X}_{t}$” in the ITS regression model. But it will lead to an erroneous result for the main effects of the level change. Fourth, the consideration of time-varying confounding is lacking. Participants-level confounding should be considered and controlled if the population was changed at each time point [6, 19]. Fifth, most of the included studies ignored the hierarchical data structure and aggregated the outcome to the population level, even if they had the opportunity to aggregate the outcome at a lower level. As we discussed above, when the intervention is implemented regionwide or nationwide, the dataset may contain a hierarchical structure. If the outcome is aggregated at a higher level, which does not account for the heterogeneity among patients and across hospitals, it will lead to aggregation bias [18, 19, 25, 32].

### Comparison with other studies

Several studies have systematically reviewed methodological issues regarding the design and statistical analysis of the ITS study [11, 21–25]. All of the previous reviews pointed out that the considerations of autocorrelation, non-stationary and seasonality were limited, which was aligned with our study. Five of them reported the sample size considerations which focused on the minimization of data points, while our study also pointed out that the maximum of data points should also be a consideration.

Some methodological issues have been improved among the ITS studies published in 2021. For example, for the item “clearly segment time”, the reported proportion has seen a notable increase, rising from 84.5% (as observed in Jandoc et al.'s review) to 100% in our study. However, some issues remain a concern (e.g., sample size, missing data, incorrect interpretation of level change due to time parameterization, time-varying participants-level confounding, and data hierarchical structure). A previous review that included a meta-analysis and re-analysis of ITS studies found that 5% (2/41) of studies did not report the statistical method used [33]. In our review, this proportion is 15.0% (23/153), indicating a higher proportion of inadequate reporting in original articles.

Our study gave a detailed analysis of three ever ignored but important methodological issues, including common errors in parameter interpretation of ITS models, limited consideration of individual-level characteristics and poor handling of heterogeneous data among clusters. Although a methodology study published in 2020 pointed out this problem and a corrigendum to the original tutorial had been made [17, 34], this was still a common mistake in ITS empirical studies published in 2021. Individual-level characteristic is also an important issue. If patient characteristics vary over time, it is essential to control for these changes using appropriate methods. For the potential cluster effects, our result showed that most of the studies had the opportunity to control the potential heterogeneity from different clusters, but few of them considered it.

### Strengths and limitations

This study gives a comprehensive survey of the methodological issues in the design and statistical analysis of ITS studies in drug utilization. To the best of our knowledge, this is the first cross-sectional survey that exclusively assesses the incorrect interpretation of level change due to time parameterization, time-varying individual-level covariates and handling of hierarchical data in current ITS studies, which have been highlighted in the methodological literature. Meanwhile, we updated the current practices of ITS in the field of drug utilization research. ITS is a frequently used method in evaluating a population-level intervention, and there is a series of literature on methodological considerations published over the past few years. It is worth analyzing and showing the limitations in methodological issues of ITS practices.

There are also three limitations in our studies. First, we only included the ITS studies published in 2021 and used a single database for searching. However, since PubMed contains nearly all healthcare science & service and public health research journals, we think that it can represent the current practices sufficiently. Another limitation is that we assess the design and statistical characteristics through the reporting of the article. If the reporting of these aspects is insufficient, we cannot determine the items and the results may be inaccurate. Third, some items may not be relevant to all studies. For example, in ITS studies using aggregated data, authors might not be able to assess the proportion of missing data at the individual level. Consequently, they may not report missing data in their articles.

## Conclusion

In summary, we identified a series of deficiencies in design and statistical analysis among current ITS studies, showing that the basic methodological issues are not improved, and some new issues are not widely considered (i.e., incorrect interpretation of level change due to time parameterization, time-varying individual characteristics and hierarchical data structure). Although a series of methodology reviews and tutorials mentioned the important issues in ITS design, there is still a significant gap between guidelines and practices of ITS studies in drug utilization research, accentuating that it is need to develop a clearer guide and checklist for conducting ITS study.

### Supplementary Information

**Supplementary Material 1**.

## Data Availability

Details of the search strategy and included studies are in the appendix.

## Abbreviations

ITS: Interrupted time series
OLS: Ordinary least square
ARIMA: Autoregressive integrated moving average model
GLS: Generalized least square
IQR: Interquartile range
