# Supplementary material for: Design and statistical analysis reporting among interrupted time series studies in drug utilization research: a cross-sectional survey
Source: BMC Med Res Methodol. 2024 Mar 9;24:62. doi: 10.1186/s12874-024-02184-8 (PMC10924989; doi:10.1186/s12874-024-02184-8)
Supplement: Supplementary file 1 — Supplementary Material 1. [file 12874_2024_2184_MOESM1_ESM.docx]

**Supplementary Material**

Contents

[Appendix 1. The parameter setting of statistical model 2](#_Toc155355020)

[Appendix 2. Search strategy of our study 3](#_Toc155355021)

[Appendix 3. Summary of the data extraction form 4](#_Toc155355022)

[Appendix 4. Included ITS studies in our survey 6](#_Toc155355023)

Appendix 1. The parameter setting of statistical model

Equation (1) shows the standard ITS model. In this model, Yt represents the outcomes at time point t, Tt represents the time variable in ITS analysis, with the first time point of the study period being 1 and so forth. Xt indicates whether the policy was implemented; Xt = 0 before implementation, and Xt = 1 after implementation. T0 is the starting point of the intervention. β0 is the intercept, β1 is the slope before intervention implementation (underlying pre-intervention trend), β2 represents the immediate level (or intercept) change immediately following the intervention, and β3 is interpreted as the change in the slope of the trend following the intervention, compared with the pre-intervention trend.

$Y_{t}=\beta_{0}+\beta_{1}T_{t}+\beta_{2}X_{t}+\beta_{3}\left( T_{t}-T_{0} \right)\cdot X_{t}$ (1)

The “wrong” model setting is the equation (2), and we can rearrange this equation as:

$$Y_{t}{=\beta}_{0}'+\beta_{1}'T_{t}+\beta_{2}'X_{t}+\beta_{3}'T_{t}\cdot X_{t}$$

$${=\beta}_{0}'+\beta_{1}'T_{t}+\beta_{2}'X_{t}+\beta_{3}'{[\left( T_{t}-T_{0} \right)+T}_{0}]\cdot X_{t}$$

${=\beta}_{0}'+\beta_{1}'T_{t}+{(\beta}_{2}'{+\beta_{3}'T_{0})X}_{t}+\beta_{3}'\left( T_{t}-T_{0} \right)\cdot X_{t}$ (2)

Therefore, the estimated β0’, β1’, and β3’ from equation (2) are equivalent to the estimated β0, β1, and β3 respectively from equation (1). But the β2 should be equal to β2’+ β3’T0, indicating that if the studies use equation (2) as the ITS model, they will get a wrong estimation of β2.

Appendix 2. Search strategy of our study

| **No.** | **Query** | **Results** |
| --- | --- | --- |
| **1** | interrupted time series analysis[MeSH Terms] | 1,494 |
| **2** | interrupted time series[Title/Abstract] | 4,302 |
| **3** | change point[Title/Abstract] | 1,126 |
| **4** | segmented regression[Title/Abstract] | 849 |
| **5** | segmented linear regression[Title/Abstract] | 199 |
| **6** | repeated measures study[Title/Abstract] | 786 |
| **7** | piecewise regression[Title/Abstract] | 244 |
| **8** | time series intervention[Title/Abstract] | 45 |
| **9** | phase design[Title/Abstract] | 394 |
| **10** | multiple baseline[Title/Abstract] | 2,206 |
| **11** | ARIMA[Title/Abstract] | 1,354 |
| **12** | integrated moving average[Title/Abstract] | 1,255 |
| **13** | 1 OR 2 OR 3 OR 4 OR 5 OR 6 OR 7 OR 8 OR 9 OR 10 OR 11 OR 12 | 11,614 |
| **14** | Limit 13 to English language | 11,441 |
| **15** | Limit 14 to 2021 | 1,862 |

Appendix 3. Summary of the data extraction form

| Category | Items |
| --- | --- |
| **General characteristics** | Title, journal, first author, study sites, data source (e.g. hospital data, insurance data), intervention stages (single-stage intervention or multi-stage intervention) ^a^, type of intervention, level of intervention (e.g., hospital-level, national-level), measure type of outcome (e.g., drug utilization, health outcome) ^b^, data type of outcome (e.g., rate, continues) |
| **Design** | 1. **Rationale for ITS**   Reason for using ITS (e.g., natural experiment, no adequate group), clearly segment time, use of control group, type of control (e.g., characteristic, location, historical, outcome)   1. **Data collection and aggregation**   Data collection (prospectively or retrospectively), raw data (if the studies contained individual-level data), analysis unit (individual unit or aggregated unit), time intervals (e.g. monthly, yearly) and the number of time points   1. **Setting the ITS impact model**   ITS impact model (e.g., both level and slope change), allow for delay impact |
| **Statistical analysis** | 1. **Basic statistical analysis characteristics**   Regression model (e.g., OLS, GLS, ARIMA), additional time trends, missing data, outliers, sensitivity analysis, statistical software ^c^, data and code available   1. **Basic methodological considerations**  - Autocorrelation - Non-stationarity - Seasonality  1. **Additional methodological considerations**  - Parameters setting (if the study reported the regression model and interpreted the coefficients; if it was right) - Individual-level covariates (if the study contained individual-level data; if it controlled individual characteristics) - Hierarchical data structure (data structure for ITS analysis; if the author handled hierarchical data; methods for handling hierarchical data; considered cluster effects in which level) |

Notes:

1. Intervention: If the study contained more than one intervention, we regarded the first intervention mentioned in the article as the intervention we were interested in.
2. Outcome: If the study contained more than one outcome, we regarded the primary outcome as the outcome we were interested in. If the author did not define the primary outcome, we regarded the first outcome mentioned in the article as the outcome we were interested in.
3. Statistical software: if the study reported that more than one statistical software was used, we collected the first statistical software reported in the article.

Appendix 4. Included ITS studies in our survey

| ID | Title | First author | Journal |
| --- | --- | --- | --- |
| 1 | A Resident-Led Intervention to Increase Initiation of Buprenorphine Maintenance for Hospitalized Patients With Opioid Use Disorder | Thakrar AP | J Hosp Med |
| 2 | A drug-related Good Samaritan Law and calling emergency medical services for drug overdoses in a Canadian setting | Moallef S | Harm Reduct J |
| 3 | Abortion Safety and Use with Normally Prescribed Mifepristone in Canada | Schummers L | N Engl J Med |
| 4 | Academic Detailing to Reduce Sedative-Hypnotic Prescribing in Older Veterans | Ragan AP | J Pharm Pract |
| 5 | Accessibility to Medication for Opioid Use Disorder After Interventions to Improve Prescribing Among Nonaddiction Clinics in the US Veterans Health Care System | Hawkins EJ | JAMA Netw Open |
| 6 | Administration of epinephrine by advanced emergency medical technicians for out-of-hospital cardiac arrest in a rural emergency medical services system | Bomba JJ | J Am Coll Emerg Physicians Open |
| 7 | Ambulatory Fluoroquinolone Use in the United States, 2015-2019 | Umarje SP | Open Forum Infect Dis |
| 8 | An Interrupted Time-series Evaluation of the Association Between State Laws Mandating Prescriber Use of Prescription Drug Monitoring Programs and Discontinuation of Chronic Opioid Therapy in US Veterans | Arnold J | Med Care |
| 9 | Assessing local California trends in emergency physician opioid prescriptions from 2012 to 2020: Experiences in a large academic health system | Elder JW | Am J Emerg Med |
| 10 | Assessing the impact of a restrictive opioid prescribing law in West Virginia | Sedney CL | Subst Abuse Treat Prev Policy |
| 11 | Assessment of a quality improvement intervention to decrease opioid prescribing in a regional health system | Brown CS | BMJ Qual Saf |
| 12 | Association Between State Policies on Improving Opioid Prescribing in 2 States and Opioid Overdose Rates Among Reproductive-aged Women | Ji X | Med Care |
| 13 | Association Between Statewide Opioid Prescribing Interventions and Opioid Prescribing Patterns in North Carolina, 2006-2018 | Maierhofer CN | Pain Med |
| 14 | Association of 3 CDC opioid prescription guidelines for chronic pain and 2 payer pharmacy coverage changes on opioid initiation practices | Togun AT | J Manag Care Spec Pharm |
| 15 | Association of Fluoroquinolone Prescribing Rates With Black Box Warnings from the US Food and Drug Administration | Sankar A | JAMA Netw Open |
| 16 | Association of US Food and Drug Administration Removal of Indications for Use of Oral Quinolones With Prescribing Trends | Tran PT | JAMA Intern Med |
| 17 | Association of the 2016 US Centers for Disease Control and Prevention Opioid Prescribing Guideline With Changes in Opioid Dispensing After Surgery | Sutherland TN | JAMA Netw Open |
| 18 | CD4+ T-cell count at antiretroviral therapy initiation in the "treat all" era in rural South Africa: an interrupted time series analysis | Yapa HM | Clin Infect Dis |
| 19 | CancelRx: a health IT tool to reduce medication discrepancies in the outpatient setting | Watterson TL | J Am Med Inform Assoc |
| 20 | Changes in Drug Utilization After Publication of Clinical Trials and Drug-Related Scandals in Japan: An Interrupted Time Series Analysis, 2005-2017 | Fukuma S | J Epidemiol |
| 21 | Changes in Erythropoiesis Stimulating Agent Use Under a Risk Evaluation and Mitigation Strategy (REMS) Program | Sarpatwari A | Drug Saf |
| 22 | Changes in Healthcare Resource Use and Costs in Commercially Insured Insomnia Patients Initiating Suvorexant | Kale HP | Adv Ther |
| 23 | Changes in Opioid Prescribing Following the Implementation of State Policies Limiting Morphine Equivalent Daily Dose in a Commercially Insured Population | Heins SE | Med Care |
| 24 | Changes in Prescribing by Provider Type Following a State Prescription Opioid Restriction Law | Valdes IL | J Gen Intern Med |
| 25 | Changes in Quantity of Opioids Dispensed following Florida's Restriction Law for Acute Pain Prescriptions | Hincapie-Castillo JM | Pain Med |
| 26 | Changes in antibiotic consumption, AMR and Clostridioides difficile infections in a large tertiary-care center following the implementation of institution-specific guidelines for antimicrobial therapy: A nine-year interrupted time series study | Schönherr SG | PLoS One |
| 27 | Changes in early high-risk opioid prescribing practices after policy interventions in Washington State | Sears JM | Health Serv Res |
| 28 | Changes in prescribing rates of sodium-containing medications in the UK from 2009 to 2018: a cross-sectional study with interrupted time series analysis | Ju C | BMJ Open |
| 29 | Chronic Opioid Therapy Utilization Following an Acute Pain Prescription Supply Restriction Law: An Interrupted Time Series Analysis | Shen Y | Pain Physician |
| 30 | Delivery-based criteria for empiric antibiotic administration among preterm infants | Garber SJ | J Perinatol |
| 31 | Development and Impact of an Institutional Enhanced Recovery Program on Opioid Use, Length of Stay, and Hospital Costs Within an Academic Medical Center: A Cohort Analysis of 7774 Patients | Thiele RH | Anesth Analg |
| 32 | Does Etanercept Biosimilar Prescription in a Rheumatology Center Bend the Medication Cost Curve? | Müskens WD | J Rheumatol |
| 33 | Does additional monitoring status increase the reporting of adverse drug reactions? An interrupted time series analysis of EudraVigilance data | Segec A | Pharmacoepidemiol Drug Saf |
| 34 | Does price deregulation increase drug price in China? An interrupted time series analysis | Xiong Y | Int J Health Plann Manage |
| 35 | Effect of a Prescription Drug Monitoring Program on Emergency Department Opioid Prescribing | Gupta R | West J Emerg Med |
| 36 | Effect of an Electronic Health Record "Nudge" on Opioid Prescribing and Electronic Health Record Keystrokes in Ambulatory Care | Ancker JS | J Gen Intern Med |
| 37 | Effect of an Intensified Antibiotic Stewardship Program at an Orthopedic Surgery Department | Feihl S | Surg Infect (Larchmt) |
| 38 | Effect of an Online Reimbursement Application System on Prescribing of Lidocaine 5% Medicated Plaster in the Republic of Ireland | Smith A | Appl Health Econ Health Policy |
| 39 | Effect of antibiotic stewardship interventions in primary care on antimicrobial resistance of Escherichia coli bacteraemia in England (2013-18): a quasi-experimental, ecological, data linkage study | Aliabadi S | Lancet Infect Dis |
| 40 | Effect of electronic medication reconciliation at the time of hospital discharge on inappropriate medication use in the community: an interrupted time-series analysis | Welk B | CMAJ Open |
| 41 | Effect of healthcare system reforms on public hospitals' revenue structures: Evidence from Beijing, China | Gao L | Soc Sci Med |
| 42 | Effectiveness of peer-supervision on pediatric fever illness treatment among registered private drug sellers in East-Central Uganda: An interrupted time series analysis | Bagonza A | Health Sci Rep |
| 43 | Effects of Chinese medical pricing reform on the structure of hospital revenue and healthcare expenditure in county hospital: an interrupted time series analysis | Liu M | BMC Health Serv Res |
| 44 | Effects of Regulation on Carbapenem Prescription in a Large Teaching Hospital in China: An Interrupted Time Series Analysis, 2016-2018 | Xie L | Infect Drug Resist |
| 45 | Effects of State Law Limiting Postoperative Opioid Prescription in Patients After Cesarean Delivery | Potnuru PP | Anesth Analg |
| 46 | Effects of a multifaceted intervention to promote the use of intravenous iron sucrose complex instead of ferric carboxymaltose in patients admitted for more than 24 h | Touchard J | Eur J Clin Pharmacol |
| 47 | Effects of a national policy advocating rational drug use on decreases in outpatient antibiotic prescribing rates in Thailand | Waleekhachonloet O | Pharm Pract (Granada) |
| 48 | Effects of a nudge-based antimicrobial stewardship program in a pediatric primary emergency medical center | Shishido A | Eur J Pediatr |
| 49 | Effects of medical service fee revision on reducing irrational psychotropic polypharmacy in Japan: an interrupted time-series analysis | Okada Y | Soc Psychiatry Psychiatr Epidemiol |
| 50 | Effects of the FDA Codeine Safety Investigation on Racial and Geographic Disparities in Opioid Prescribing after Pediatric Tonsillectomy and/or Adenoidectomy | Lawrence A | Glob Pediatr Health |
| 51 | Enhanced recovery after surgery protocol and postoperative opioid prescribing for cesarean delivery: an interrupted time series analysis | Langnas EM | Perioper Med (Lond) |
| 52 | Evaluation of a Pilot Vancomycin Precision Dosing Advisory Service on Target Exposure Attainment Using an Interrupted Time Series Analysis | Stocker SL | Clin Pharmacol Ther |
| 53 | Evaluation of a quality improvement bundle aimed to reduce opioid prescriptions after Cesarean delivery: an interrupted time series study | Laksono I | Can J Anaesth |
| 54 | Extent of a valsartan drug shortage and its effect on antihypertensive drug use in the Canadian population: a national cross-sectional study | Fenna J | CMAJ Open |
| 55 | Guidance impact on primary care prescribing rates of simple analgesia: an interrupted time series analysis in England | Reichel H | Br J Gen Pract |
| 56 | How government health insurance coverage of novel anti-cancer medicines benefited patients in China - a retrospective analysis of hospital clinical data | Diao Y | BMC Health Serv Res |
| 57 | Ignoring instead of chasing after coagulation factor VII during warfarin management: an interrupted time series study | Oskarsdottir AR | Blood |
| 58 | Impact of Check of Medication Appropriateness (CMA) in optimizing analgesic prescribing: An interrupted time series analysis | Quintens C | Eur J Pain |
| 59 | Impact of China's zero mark-up drug policy on drug cost of NCDs' outpatients: an interrupted time series analysis | Du J | BMC Health Serv Res |
| 60 | Impact of Clinical Practice Guidelines on Blood Glucose Test Strip Prescription Rates in Manitoba and Saskatchewan (Canada): An Interrupted Time-Series Analysis | Nichols J | Can J Diabetes |
| 61 | Impact of EMA regulatory label changes on hydroxyzine initiation, discontinuation and switching to other medicines in Denmark, Scotland, England and the Netherlands: An interrupted time series regression analysis | Morales DR | Pharmacoepidemiol Drug Saf |
| 62 | Impact of EU regulatory label changes for diclofenac in people with cardiovascular disease in four countries: Interrupted time series regression analysis | Morales DR | Br J Clin Pharmacol |
| 63 | Impact of Generic Entry on Hospital Antimicrobial Use: A Retrospective Quasi-Experimental Interrupted Time Series Analysis | Espona M | Antibiotics (Basel) |
| 64 | Impact of Medicaid Eligibility Changes on Long-acting Reversible Contraception Use in Massachusetts and Maine | Pace LE | Med Care |
| 65 | Impact of Medicare prescription drug (Part D) coverage expansion on utilisation and financial burden of benzodiazepines among older adults: an interrupted time series analysis | Li M | BMJ Open |
| 66 | Impact of Opioid Restrictions During a Critical Drug Shortage Period: Interrupted Time Series for Institutional Opioid Utilization | Brokenshire SA | Pain Med |
| 67 | Impact of Revised Infectious Diseases Society of America and Society for Healthcare Epidemiology of America Clinical Practice Guidelines on the Treatment of Clostridium difficile Infections in the United States | Clancy CJ | Clin Infect Dis |
| 68 | Impact of Vonoprazan Triple-Drug Blister Packs on H. pylori Eradication Rates in Japan: Interrupted Time Series Analysis | Deguchi H | Adv Ther |
| 69 | Impact of a Mandatory Prescription Drug Monitoring Program Check on Emergency Department Opioid Prescribing Rates | Watson CJ | J Med Toxicol |
| 70 | Impact of a Mortality Prediction Rule for Organizing and Guiding Antimicrobial Stewardship Program Activities | Collins CD | Open Forum Infect Dis |
| 71 | Impact of a Social Marketing Intervention on General Practitioners' Antibiotic Prescribing Practices for Acute Respiratory Tract Complaints in Malta | Machowska A | Antibiotics (Basel) |
| 72 | Impact of a drug safety communication on the severity of benzonatate exposures reported to poison centers | Leonard JB | Pharmacoepidemiol Drug Saf |
| 73 | Impact of a hospital-wide computerised approach to optimise the quality of antimicrobial prescriptions in patients with severe obesity: a quasi-experimental study | Sirard S | BMC Infect Dis |
| 74 | Impact of a nationwide prospective drug utilization review program to improve prescribing safety of potentially inappropriate medications in older adults: An interrupted time series with segmented regression analysis | Jang S | Pharmacoepidemiol Drug Saf |
| 75 | Impact of a non-compulsory antifungal stewardship program on overuse and misuse of antifungal agents in a tertiary care hospital | Markogiannakis A | Int J Antimicrob Agents |
| 76 | Impact of a policy change restricting access to codeine on prescription opioid-related emergency department presentations: an interrupted time series analysis | Elphinston RA | Pain |
| 77 | Impact of an Antibiotic Stewardship Program on the Incidence of Resistant Escherichia coli: A Quasi-Experimental Study | Ziv-On E | Antibiotics (Basel) |
| 78 | Impact of an Antimicrobial Stewardship Program on the Incidence of Carbapenem Resistant Gram-Negative Bacilli: An Interrupted Time-Series Analysis | López-Viñau T | Antibiotics (Basel) |
| 79 | Impact of an electronic alert on prescription patterns of meropenem, voriconazole and caspofungin | Chok L | BMC Infect Dis |
| 80 | Impact of cascade reporting of antimicrobial susceptibility on fluoroquinolone and meropenem consumption at a Veterans' Affairs medical center | Vissichelli NC | Infect Control Hosp Epidemiol |
| 81 | Impact of changes in controlled drugs legislation on benzodiazepine receptor agonist prescribing in Ireland: a repeated cross-sectional study | Cadogan CA | Eur J Clin Pharmacol |
| 82 | Impact of drug utilization management policy on prescription opioid use in Georgia Medicaid | Wang Y | J Pharm Health Serv Res |
| 83 | Impact of national guidelines for antimicrobial stewardship to reduce antibiotic use in upper respiratory tract infection and gastroenteritis | Sato D | Infect Control Hosp Epidemiol |
| 84 | Impact of policy changes on the provision of naloxone by pharmacies in Ontario, Canada: a population-based time-series analysis | Antoniou T | Addiction |
| 85 | Impact of prescription drug monitoring program mandate on postoperative opioid prescriptions in children | Theodorou CM | Pediatr Surg Int |
| 86 | Impact of provincial and national implementation strategies on P2Y12 inhibitor utilization for acute coronary syndrome in the elderly: an interrupted time series analysis from 2008 to 2018 | Gupta S | Implement Sci |
| 87 | Impact of recommendations on sodium valproate prescription among women with epilepsy: An interrupted time-series study | Degremont A | Epilepsy Behav |
| 88 | Impact of regulatory interventions to restrict the combined use of renin-angiotensin system blockers: A Danish nationwide drug utilisation study | Sindahl P | Br J Clin Pharmacol |
| 89 | Impact of restriction of over-the-counter sales of antimicrobials on antimicrobial resistance in Escherichia coli from community-onset urinary tract infections in inner São Paulo State, Brazil | Rodrigues FS | PLoS One |
| 90 | Impact of strategic use of antiretroviral therapy intervention to the HIV continuum of care in 13 cities in Indonesia: an interrupted time series analysis | Tarigan YN | AIDS Res Ther |
| 91 | Impact of the Centers for Medicare and Medicaid Services Sepsis Core Measure on Antibiotic Use | Pakyz AL | Clin Infect Dis |
| 92 | Impact of the Low-Price Medicine Policy on Medicine Supply in China: An Interrupted Time-Series Analysis | Zhao M | Front Pharmacol |
| 93 | Impact of the National Centralized Drug Procurement Policy (4 + 7 policy) on the drug expenditures of patients treated in outpatient and emergency departments in a large tertiary level-A hospital in China: A single centre, interrupted time series | Lan T | J Clin Pharm Ther |
| 94 | Impact of the National Health Insurance Coverage Policy on the Utilisation and Accessibility of Innovative Anti-cancer Medicines in China: An Interrupted Time-Series Study | Fang W | Front Public Health |
| 95 | Impact of the introduction of falls risk assessment toolkit on falls prevention and psychotropic medicines' utilisation in Walsall: an interrupted time series analysis | Aladul MI | BMJ Open |
| 96 | Impact of the multidisciplinary antimicrobial stewardship team intervention focusing on carbapenem de-escalation: A single-centre and interrupted time series analysis | Suzuki A | Int J Clin Pract |
| 97 | Impact on In- and Outpatient Hospital Drug Prescriptions of Including a More Expensive Me-Too Antidepressant in a Hospital Drug Formulary: a Controlled Longitudinal Study | Carracedo-Martínez E | Pharmacoepidemiol Drug Saf |
| 98 | Impacts of Canada's cannabis legalization on police-reported crime among youth: early evidence | Callaghan RC | Addiction |
| 99 | Improving appropriate use of anticoagulants in hospitalised patients: a pharmacist-led Check of Medication Appropriateness intervention | Quintens C | Br J Clin Pharmacol |
| 100 | Increased Use of Complementary and Alternative Therapies for Back Pain Following Statewide Medicaid Coverage Changes in Oregon | Choo EK | J Gen Intern Med |
| 101 | Increasing Naloxone Prescribing in the Emergency Department Through Education and Electronic Medical Record Work-Aids | Funke M | Jt Comm J Qual Patient Saf |
| 102 | Influence of Chinese National Centralized Drug Procurement on the price of policy-related drugs: an interrupted time series analysis | Wang N | BMC Public Health |
| 103 | Influence of Opioid Prescription Policy on Overdoses and Related Adverse Effects in a Primary Care Population | Harder VS | J Gen Intern Med |
| 104 | Institution of prothrombin complex concentrate protocols is associated with a reduction in plasma administration at a Tertiary Care Hospital | Carabini LM | J Clin Anesth |
| 105 | Interrupted Time-Series Analysis to Evaluate the Impact of a National Antimicrobial Stewardship Campaign on Antibiotic Prescribing: A Typical Practice in China's Primary Care | Li H | Clin Infect Dis |
| 106 | Interrupted time series analysis of cannabis coding in Colorado during the ICD-10-CM transition | Hall KE | Inj Prev |
| 107 | Interrupted time series analysis to evaluate the performance of drug overdose morbidity indicators shows discontinuities across the ICD-9-CM to ICD-10-CM transition | Yang H | Inj Prev |
| 108 | Long-Term Impact of an Educational Antimicrobial Stewardship Program on Management of Patients with Hematological Diseases | Guisado-Gil AB | Antibiotics (Basel) |
| 109 | Long-term Success With Diminished Opioid Prescribing After Implementation of Standardized Postoperative Opioid Prescribing Guidelines: An Interrupted Time Series Analysis | Findlay BL | Mayo Clin Proc |
| 110 | Measuring the impacts of the Using Antibiotics Wisely campaign on Canadian community utilization of oral antibiotics for respiratory tract infections: a time-series analysis from 2015 to 2019 | Rolf von den Baumen T | J Antimicrob Chemother |
| 111 | National Trends and Policy Impacts on Provision of Home Medicines Reviews and Residential Medication Management Reviews in Older Australians, 2009-2019 | Sluggett JK | Int J Environ Res Public Health |
| 112 | Off-label postpartum use of domperidone in Canada: a multidatabase cohort study | Moriello C | CMAJ Open |
| 113 | Opioid agonist treatment uptake within provincial correctional facilities in British Columbia, Canada | Kurz M | Addiction |
| 114 | Opioid days' supply limits: an interrupted time-series analysis of opioid prescribing before and following a Massachusetts law | Hackman HH | Am J Drug Alcohol Abuse |
| 115 | Opioid dosing among patients with 3 or more years of continuous prescription opioid use before and after the CDC opioid prescribing guideline | Salas J | Int J Drug Policy |
| 116 | Opioid use in medical cannabis authorization adult patients from 2013 to 2018: Alberta, Canada | Lee C | BMC Public Health |
| 117 | Outpatient Fluoroquinolone Prescription Fills in the United States, 2014 to 2020: Assessing the Impact of Food and Drug Administration Safety Warnings | Buehrle DJ | Antimicrob Agents Chemother |
| 118 | Outpatient Fluoroquinolone Use in Children, 2000-2018 | Ross RK | J Pediatric Infect Dis Soc |
| 119 | Patterns of oxycodone controlled release use in older people with cancer following public subsidy of oxycodone/naloxone formulations: An Australian population-based study | Daniels B | Asia Pac J Clin Oncol |
| 120 | Pediatric Practice Transformation and Long-Acting Reversible Contraception (LARC) Use in Adolescents | Schiavoni KH | Acad Pediatr |
| 121 | Real-World Effectiveness of Adjuvant Oxaliplatin Chemotherapy in Stage III Colon Cancer: A Controlled Interrupted Time Series Analysis | Huang WK | Front Pharmacol |
| 122 | Reducing Duration of Antibiotic Use for Presumed Neonatal Early-Onset Sepsis in Greek NICUs. A "Low-Hanging Fruit" Approach | Kopsidas I | Antibiotics (Basel) |
| 123 | Reduction in antimicrobial use associated with a multifaceted antimicrobial stewardship programme in a tertiary teaching hospital in Shanghai: a segmented regression analysis | Liu YX | Ann Palliat Med |
| 124 | Reductions in vancomycin and meropenem following the implementation of a febrile neutropenia management algorithm in hospitalized adults: An interrupted time series analysis | Trinh TD | Infect Control Hosp Epidemiol |
| 125 | Rhode Island's Opioid Overdose Hospital Standards and Emergency Department Naloxone Distribution, Behavioral Counseling, and Referral to Treatment | Samuels EA | Ann Emerg Med |
| 126 | Short and long term impact of combining restrictive and enabling interventions to reduce aztreonam consumption in a community hospital | Mody D | Int J Clin Pharm |
| 127 | State Medical Board Policy and Opioid Prescribing: A Controlled Interrupted Time Series | Ranapurwala SI | Am J Prev Med |
| 128 | The Effects of the National Centralized Drug Purchasing Pilot Program on Nucleos(t)ide Analogs in Shenzhen City: An Interrupted Time Series Analysis | Wen X | Front Public Health |
| 129 | The Impact of CMS SEP-1 Core Measure Implementation on Antibacterial Utilization: a retrospective multicenter longitudinal cohort study with interrupted time-series analysis | Anderson DJ | Clin Infect Dis |
| 130 | The Public Health Insurance Coverage of Novel Targeted Anticancer Medicines in China-In Favor of Whom? A Retrospective Analysis of the Insurance Claim Data | Li M | Front Pharmacol |
| 131 | The children of the missed pill | Rau T | J Health Econ |
| 132 | The effect of generic market entry on antibiotic prescriptions in the United States | Kållberg C | Nat Commun |
| 133 | The effectiveness of Check of Medication Appropriateness for antimicrobial stewardship: an interrupted time series analysis | Quintens C | J Antimicrob Chemother |
| 134 | The effects of an evidence- and theory-informed feedback intervention on opioid prescribing for non-cancer pain in primary care: A controlled interrupted time series analysis | Alderson SL | PLoS Med |
| 135 | The impact of "4 + 7" volume-based drug procurement on the volume, expenditures, and daily costs of antihypertensive drugs in Shenzhen, China: an interrupted time series analysis | Yang Y | BMC Health Serv Res |
| 136 | The impact of a co-payment increase on the consumption of type 2 antidiabetics - A nationwide interrupted time series analysis | Rättö H | Health Policy |
| 137 | The impact of an outpatient parenteral antibiotic therapy (OPAT) clinic for adults with cellulitis: an interrupted time series study | Yadav K | Intern Emerg Med |
| 138 | The impact of proposed regulatory changes and rescheduling on low-dose codeine purchasing in Canada: a time-series analysis | Boone CG | CMAJ Open |
| 139 | The impact of public coverage of newer hepatitis C medications on utilization, adherence, and costs in British Columbia | Ho H | PLoS One |
| 140 | The impact of tightened prescribing restrictions on proton pump inhibitor use in Australia: An evaluation using interrupted time series analysis | Daniels B | Pharmacoepidemiol Drug Saf |
| 141 | The impacts of Chinese drug volume-based procurement policy on the use of policy-related antibiotic drugs in Shenzhen, 2018-2019: an interrupted time-series analysis | Yang Y | BMC Health Serv Res |
| 142 | The impacts of government reimbursement negotiation on targeted anticancer medication price, volume and spending in China | Zhang Y | BMJ Glob Health |
| 143 | Towards implementing an Antibiotic Stewardship Intervention (ASI) in Ecuador - Evaluating antibiotic consumption and the impact of an ASI in a tertiary hospital according to the World Health Organization (WHO) recommendations | Romo-Castillo HF | J Glob Antimicrob Resist |
| 144 | Trend changes of national zolpidem users and exposure cases after FDA drug safety communications | Zhou EH | Pharmacoepidemiol Drug Saf |
| 145 | Trends and patterns in antibiotic prescribing for adult outpatients with acute upper respiratory tract infection in Japan, 2008-2018 | Ji L | J Infect Chemother |
| 146 | Trends in Postpartum Opioid Prescribing: A Time Series Analysis | Zipursky JS | Clin Pharmacol Ther |
| 147 | Trends in hydrocodone combination product exposures reported to California Poison Control System (CPCS) following DEA rescheduling | Wu A | Clin Toxicol (Phila) |
| 148 | Trends in systemic antifungal use in Australia, 2005-2016: a time-series analysis | Wang Y | Jpn J Infect Dis |
| 149 | Trends of Antibiotic Use and Expenditure After an Intensified Antimicrobial Stewardship Policy at a 2,200-Bed Teaching Hospital in China | Qian X | Front Public Health |
| 150 | Trends of Negotiated Targeted Anticancer Medicines Use in China: An Interrupted Time Series Analysis | Huang C | Int J Health Policy Manag |
| 151 | Trends over time in drug administration during pediatric in-hospital cardiac arrest in the United States | Ross CE | Resuscitation |
| 152 | Trimetazidine Use in Parkinson's Disease: Is It a Resolved Problem? | Pintér D | eNeuro |
| 153 | Using Audit and Feedback to Improve Antimicrobial Prescribing in Emergency Departments: A Multicenter Quasi-Experimental Study in the Veterans Health Administration | Livorsi DJ | Open Forum Infect Dis |
